# Unraveling multi-fixel microstructure with tractography and angular weighting

**DOI:** 10.3389/fnins.2023.1199568

**Published:** 2023-06-07

**Authors:** Nicolas Delinte, Laurence Dricot, Benoit Macq, Claire Gosse, Marie Van Reybroeck, Gaetan Rensonnet

**Affiliations:** ^1^Institute of Information and Communication Technologies, Electronics and Applied Mathematics, Université Catholique de Louvain, Louvain-la-Neuve, Belgium; ^2^Institute of NeuroScience, Université Catholique de Louvain, Brussels, Belgium; ^3^Psychological Sciences Research Institute, Université Catholique de Louvain, Louvain-la-Neuve, Belgium

**Keywords:** diffusion MRI, white matter, tractography, microstructure, multi-fascicle models

## Abstract

Recent advances in MRI technology have enabled richer multi-shell sequences to be implemented in diffusion MRI, allowing the investigation of both the microscopic and macroscopic organization of the brain white matter and its complex network of neural fibers. The emergence of advanced diffusion models has enabled a more detailed analysis of brain microstructure by estimating the signal received from a voxel as the combination of responses from multiple fiber populations. However, disentangling the individual microstructural properties of different macroscopic white matter tracts where those pathways intersect remains a challenge. Several approaches have been developed to assign microstructural properties to macroscopic streamlines, but often present shortcomings. ROI-based heuristics rely on averages that are not tract-specific. Global methods solve a computationally-intensive global optimization but prevent the use of microstructural properties not included in the model and often require restrictive hypotheses. Other methods use atlases that might not be adequate in population studies where the shape of white matter tracts varies significantly between patients. We introduce UNRAVEL, a framework combining the microscopic and macroscopic scales to unravel multi-fixel microstructure by utilizing tractography. The framework includes commonly-used heuristics as well as a new algorithm, estimating the microstructure of a specific white matter tract with *angular weighting*. Our framework grants considerable freedom as the inputs required, a set of streamlines defining a tract and a multi-fixel diffusion model estimated in each voxel, can be defined by the user. We validate our approach on synthetic data and *in vivo* data, including a repeated scan of a subject and a population study of children with dyslexia. In each case, we compare the estimation of microstructural properties obtained with *angular weighting* to other commonly-used approaches. Our framework provides estimations of the microstructure at the streamline level, volumetric maps for visualization and mean microstructural values for the whole tract. The *angular weighting* algorithm shows increased accuracy, robustness to uncertainties in its inputs and maintains similar or better reproducibility compared to commonly-used analysis approaches. UNRAVEL will provide researchers with a flexible and open-source tool enabling them to study the microstructure of specific white matter pathways with their diffusion model of choice.

## 1. Introduction

In the field of brain research, diffusion MRI (dMRI) leveraging multi-shell sequences has emerged as an essential tool through its ability to detect changes in the microscopic and macroscopic structure of white matter which other MRI modalities are unable to capture.

At the microscopic scale, within each imaging voxel, adequate dMRI modeling estimates the orientation of axons or fascicles of axons (Tournier et al., 2007; Canales-Rodriguez et al., 2019), and finer morphological properties, referred to as *microstructure*, such as the axon diameter, axonal density or diffusivity (Alexander et al., 2019; Jelescu et al., 2020). The majority of white matter (WM) voxels contain complex crossing configurations of two or three fiber populations per voxel (Jeurissen et al., 2013), which will be referred to in this work as *fixels*, as proposed in Raffelt et al. (2015). At the macroscopic scale, tractography algorithms piece together the orientational information collected at each voxel to generate streamlines representing the course of a bundle of axons across multiple WM voxels. Tractography is a visualization tool of great interest in clinical practice, but further processing is required for quantitative analyses.

Assigning microstructural properties to macroscopic streamlines in a consistent way remains a challenging task (Assaf et al., 2013), yet it is of great interest for the study of brain structure and function, in healthy and pathological conditions (Rathi et al., 2011). It is particularly useful in population studies where the shape of WM tracts may vary significantly between patients, leading simple voxel-based comparisons to fail (Yeatman et al., 2012; Dhollander et al., 2021). By far the most common method to characterize WM tracts to date has been to rely on scalar maps of WM properties derived from single-fixel models such as in Diffusion Tensor Imaging (DTI) (Basser et al., 1994). A WM tract is characterized by averaging the microstructural properties of all voxels containing streamlines of the tract, with possible refinements such as weighting by a tract probability atlas and distance from an average streamline (Yeatman et al., 2012). The main limitation of this approach is its inability to interpret the microstructure in voxels where multiple fiber populations intersect (Jbabdi et al., 2010), although such voxels are abundant at clinical imaging resolution (Jeurissen et al., 2013; Schilling et al., 2017). Consequently, streamlines belonging to different macroscopic tracts but passing through the same voxels are inevitably assigned the same microstructural metrics. Multi-fixel models, such as Tuch et al. (2002), Scherrer et al. (2016), Fick et al. (2019), and Rensonnet et al. (2019), address the limitations of single-fixel models in areas of crossing fibers, but are more difficult to interpret and combine with macroscopic information, because of one-to-zero, one-to-one and one-to-many correspondence issues between local fixels and macroscopic tracts (Jbabdi et al., 2010; Raffelt et al., 2017; Reymbaut et al., 2021). We identify two classes of approaches to overcome these limitations: (i) microstructure-informed tractography and (ii) combining tractography information with the output of multi-fixel models.

Microstructure-informed tractography has received a lot of attention in the literature. Frameworks such as MicroTrack (Hutchison et al., 2010), SIFT (Smith et al., 2013), SIFT 2 (Smith et al., 2015), COMMIT (Daducci et al., 2015), COMMIT2 (Schiavi et al., 2020), COMMIT-*T*_2_ (Barakovic et al., 2021), COMMIT_tree (Ocampo-Pineda et al., 2021), and MesoFT (Reisert et al., 2014) use a generative signal model for each streamline, assume constant microstructure along each streamline and solve a global optimization over the whole WM to simultaneously filter streamlines and estimate their microstructural properties. These methods may be limited in the range of microstructural parameters which can be assigned to WM tracts: SIFT and SIFT 2 are mainly designed to estimate fiber volume and density while COMMIT methods estimate the diameter of each streamline and may not enable the estimation of more phenomenological properties such as diffusivities. AxTract (Girard et al., 2017) relaxes the hypothesis of constant microstructure along streamlines but requires a multi-fixel model with an estimate of the axon diameter for each fixel, which is challenging with current acquisition protocols. Recently, an extension of the COMMIT framework was proposed to estimate the myelin content of crossing streamlines separately from a scalar map of voxel-wise myelin content (Schiavi et al., 2022).

Fewer approaches have been proposed to combine multi-fixel models with tractography. Connectivity-based fixel enhancement (CFE) (Raffelt et al., 2015) and a fixel-based analysis (FBA) framework (Raffelt et al., 2017; Dhollander et al., 2021) were proposed for group comparisons of fixel-specific measures across the white matter, wherein fixel-specific metrics are smoothed only with the fixels sharing common streamlines, with a focus on axon density. However, a challenging step of this method is the creation of a group-averaged template of Fiber Orientation Distributions (FODs). This may introduce distortions, artifacts or excessive smoothing when the brain morphology presents abnormalities. Furthermore, a streamline segment in a voxel is only assigned the metrics of the fixel with the closest orientation, which does not allow multiple local fixels to contribute to a given streamline. This “closest-fixel-only” strategy was also used in Rensonnet et al. (2019) and Reymbaut et al. (2021) when analyzing the microstructural properties of macrostructural WM tracts using a multi-fixel model.

This work focuses on the latter class of approaches, i.e., the combination of multi-fixel models and tractography, and introduces a framework named UNRAVEL. The only inputs required for our framework are any choice of multi-fixel microstructural model and a set of streamlines specific to a macroscopic tract of interest, which can be generated by any tractography algorithm and isolated with any method (Wassermann et al., 2016). The streamlines can be generated independently and do not need to match the orientations of the fixels in the microstructural model. We propose a lightweight framework relating streamline segments to local fixels, which includes the closest-fixel-only and a proposed *angular weighting* strategy among various options. Our framework also allows microstructural properties to vary along the course of an individual streamline. We provide theoretical interpretations at the tract and at the streamline segment level, which enables UNRAVEL to estimate the microstructure at the streamline level as well as mean microstructural values for a whole tract. We validate the method on a synthetic phantom, on a scan-rescan experiment on a healthy adult, as well as on a small population of children with dyslexia and control children.

## 2. Theory

**Figure 1:**
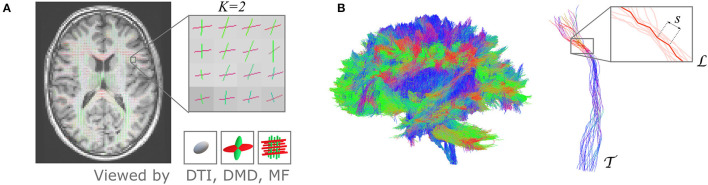
The proposed UNRAVEL framework requires two independent inputs: a multi-fixel microstructural model estimated in the white matter and the streamlines of a given macroscopic tract of interest. **(A)** 2D slice of a volume with up to *K* fixels in each voxel obtained with a multi-fixel model (such as DIAMOND [DMD] and Microstructure Fingerprinting [MF]), with each fixel possessing a main orientation (shown as colored sticks) and different microstructural properties. **(B)** Illustration of a macroscopic tract T, composed of streamlines L made of segments *s*. In this illustration, tract T was isolated from a set of whole-brain tractography streamlines.

The UNRAVEL framework requires two inputs for each subject, schematically illustrated in Figure 1. First, an estimation of a *K*-fixel model in every voxel *v* of the WM, in which every fixel *k* is characterized by a principal orientation ${û}_{vk}^{\mu }$ and fixel-specific microstructural metrics $M_{vk}^{\mu }$, *k* = 1, …, *K*, where typically *K* = 2 or 3. Second, a macroscopic tract T, defined as a set of streamlines, based on anatomical or functional relevance. Each streamline L is composed of small, straight segments *s*, with a length equal to the step size parameter in the tractography.

### 2.1. The UNRAVEL framework

The main concept behind UNRAVEL is to treat each streamline segment *s* of a macroscopic tract T individually and assign each segment microstructural properties in each voxel *v* based on the fixels in that voxel. The key quantity to achieve this is defined below.

#### 2.1.1. Relative contribution of a fixel to a streamline segment

The relative contribution of fixel *k* to a streamline segment *s* in voxel *v* is denoted α_*vsk*_ and must satisfy


(1) $$\begin{array}{l} \alpha_{vsk}\in \left[0,1\right]\\ \sum_{k=1}^{K}\alpha_{vsk}\;=1\;\forall \;v,s. \end{array}$$

Streamline segments crossing voxels boundaries are divided into smaller subsegments, each of which is enclosed within a single voxel and is then processed individually. Three definitions are considered below, referred to as relative volume weighting, closest-fixel-only and angular weighting. In contrast to the relative volume weighting approach, which is independent of the angle between the segment *s* and the fixel *k*, closest-fixel-only and angular weighting are determined by the angular difference between the two.

##### 2.1.1.1. Relative volume weighting (*vol*)

This method attributes a relative contribution using the relative volume fraction of each fixel in a voxel *v*


(2) $$\alpha_{vsk}=\frac{f_{vk}}{\sum_{k}f_{vk}},$$

where *f*_*vk*_ is the volume fraction of fixel *k* estimated by the multi-fixel model. The resulting relative contribution is not dependent on *s*. In the absence of an isotropic compartment, this equation can be simplified to α_*vsk*_ = *f*_*vk*_ as the volume fraction of each fixel sum to one, as in Ahmed Sid et al. (2017).

##### 2.1.1.2. Closest-fixel-only (*cfo*)

A segment receives a contribution from a single fixel in the voxel, based on the angular distance. The fixel *k* with orientation ${û}_{vk}^{\mu }$ closest to the orientation **û**_*vs*_ of a streamline segment *s* in voxel *v* gives its properties to segment *s* while the other fixels do not contribute. This is the most commonly used strategy in methods combining microstructure and tractography such as MicroTrack (Hutchison et al., 2010), CFE (Raffelt et al., 2015), and Magic DIAMOND (Reymbaut et al., 2021). Mathematically, for *k* = 1, …, *K*,


(3) $$\alpha_{vsk}\;=\left\{ \begin{array}{l} 1\text{ if }k=\underset{k^{\prime }}{\text{argmin }}∠{\hat{u}}_{vs},{\hat{u}}_{vk^{\prime }}^{\mu }\\ \;0\text{ elsewhere}, \end{array}\right.$$

where ∠a, b denotes the angle between vectors a and b.

##### 2.1.1.3. Angular weighting (*ang*)

A relative contribution α_*vsk*_ is assigned to all fixels *k* in a voxel *v* based on the relative angle difference between the fixels and the orientation of the streamline segment *s*. The closer a fiber population orientation is to the orientation of the segment, the closer α_*vsk*_ is to 1 and the more this fixel contributes to the microstructural properties assigned to *s*. Mathematically, for *k* = 1, …, *K*,


(4) $$\begin{array}{l} \phi =\min(90,\sum_{k^{\prime }=1}^{K}∠{\hat{u}}_{vs},{\hat{u}}_{vk^{\prime }}^{\mu })\\ \alpha_{vsk}=\{\begin{array}{ll} \frac{\phi -∠{\hat{u}}_{vs},{\hat{u}}_{vk}^{\mu }}{\phi \cdot K-\sum_{k^{\prime }=1}^{K}∠{\hat{u}}_{vs},{\hat{u}}_{vk^{\prime }}^{\mu }} & \text{ for}K>1,\\ 1 & \text{ for}K=1. \end{array} \end{array}$$

This definition is expected to be useful in tracts T in which axons exhibit microscopic dispersion (Nilsson et al., 2012; Mollink et al., 2017) where multiple fixels may be required to explain the signal. It also captures the stochastic nature of the streamline segment orientation in probabilistic tractography.

**Figure 2:**
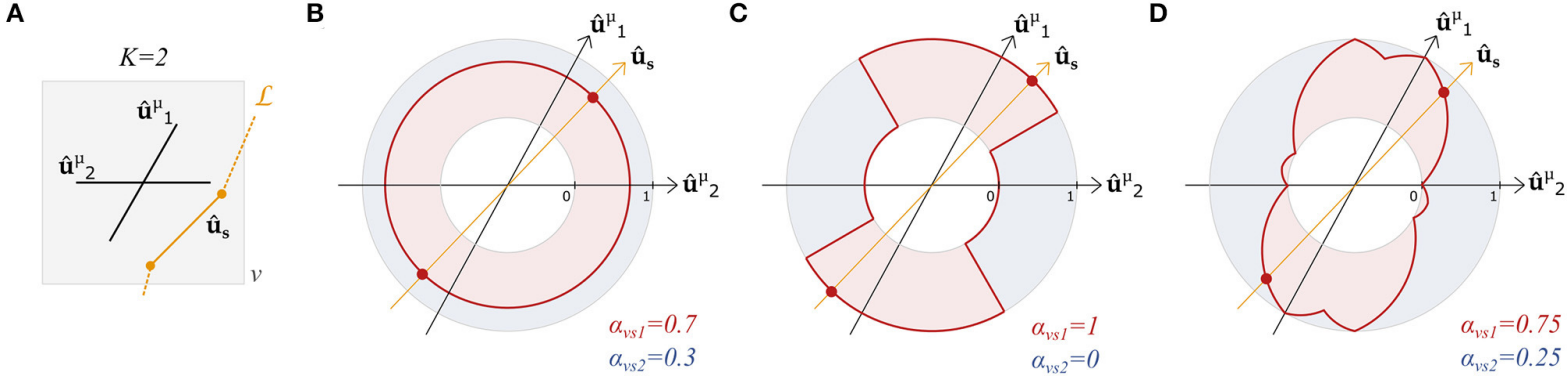
Angular weighting attributes a relative weight to all fixels based on the angle difference. Graphical representation of **(A)** the orientations ${û}_{1}^{\mu }$ and ${û}_{2}^{\mu }$ of two fixels and a segment *s* of a streamline L in a voxel *v*. Relative contribution α_*vsk*_ of fixel 1 (*k* = 1, red) and fixel 2 (*k* = 2, blue) with **(B)** relative volume weighting, **(C)** closest-fixel-only, and **(D)** angular weighting strategies as a function of the orientation **û**_*s*_ of the streamline segment in the voxel.

Figure 2 graphically compares the above definitions of α_*vsk*_ in a case with *K* = 2 fixels in a voxel. With these definitions, streamline- and tract-specific maps and metrics can now be defined.

#### 2.1.2. Streamline microstructure

For all segments *s* of a given streamline, the segment-specific microstructural metric *M*_*vs*_ is defined in voxel *v* as


(5) $$\begin{array}{l} M_{vs}=\overset{K}{\underset{k=1}{\sum }}\alpha_{vsk}M_{vk}^{\mu }. \end{array}$$

This value varies with *s*, which allows a streamline to have non-constant microstructure along its course.

#### 2.1.3. Fixel weight maps

We first define the segment-specific fixel weight *w*_*vsk*_ as


(6) $$\begin{array}{l} w_{vsk}=\alpha_{vsk}l_{vs}, \end{array}$$

where *l*_*vs*_ is the length of segment *s*, restricted to voxel *v* if segment *s* spans multiple voxels. For each fixel *k*, a fixel weight map $w_{vk}^{T}$ of the streamline segments *s* in tract T is then defined as


(7) $$\begin{array}{l} w_{vk}^{T}=\underset{s}{\sum }w_{vsk}=\underset{s}{\sum }\alpha_{vsk}l_{vs}. \end{array}$$

Such a map shows in each voxel *v* the importance of fixel *k* in assigning microstructural properties to the segments of tract T, with higher weight associated to longer and more numerous streamline segments in that voxel and to higher relative contribution α_*vsk*_ of fixel *k*. Note that this map does *not* exhibit spatial smoothness in general because the *k*-th fixel of one voxel may not correspond to the same macroscopic tract T as the *k*-th fixel in neighboring voxels.

Finally, the summation of Equation (7) over *k* gives the map of total segment lengths $w_{v}^{T}$ in each voxel [using Equation (1) for the last equality]


(8) $$\begin{array}{l} w_{v}^{T}=\overset{K}{\underset{k=1}{\sum }}w_{vk}^{T}=\overset{K}{\underset{k=1}{\sum }}\underset{s}{\sum }\alpha_{vsk}l_{vs}=\underset{s}{\sum }l_{vs}, \end{array}$$

which does not depend on the fixels' microstructural properties and is entirely determined by the tractography. This map will generally exhibit spatial smoothness and can be interpreted as the probability of the presence of tract T.

#### 2.1.4. Microstructure maps

Summing the fixel-specific microstructural metrics $M_{vk}^{\mu }$ provided as input to our method weighted by the above-defined fixel weight maps produces the following map


(9) $$M_{v}^{T}=\;\frac{\sum_{k=1}^{K}w_{vk}^{T}M_{vk}^{\mu }}{\sum_{k=1}^{K}w_{vk}^{T}}$$

which gives an average microstructural metric in each voxel *v* representing all the streamlines of tract T. This map exhibits more spatial smoothness and facilitates visualization of tract microstructure. The confidence level of its values can be guided by the segment lengths map defined in Equation (8) above.

#### 2.1.5. Mean tract microstructural metric

An overall scalar summary ${\bar{M}}^{T}$ of a specific microstructural metric *M*^μ^ for tract T can be defined using Equation (9) and a weighted map $\gamma_{v}^{T}$, specifying the respective weights of each voxels for the mean value


(10) $${\bar{M}}^{T}=\frac{\sum_{v}\gamma_{v}^{T}M_{v}^{T}}{\sum_{v}\gamma_{v}^{T}}.$$

Two definitions of weighted map $\gamma_{v}^{T}$ are considered below, referred to as total segment length and region of interest weighting.

##### 2.1.5.1. Total segment length weighting (*tsl*)

A first weighted map can be defined using the total segment length $w_{v}^{T}$ defined in Equation (8), where voxels with a high fixel weight contribute more to the final metric.


(11) $$\begin{array}{l} \gamma_{v}^{T}=w_{v}^{T}. \end{array}$$

##### 2.1.5.2. Region of interest weighting (*roi*)

Another weighted map can be defined by attributing equal weights to all voxels *v* contained in the tract.


(12) $$\gamma_{v}^{T}=\left\{ \begin{array}{l} 1\;\forall \;v\in T\\ 0\text{ elsewhere}. \end{array}\right.$$

### 2.2. Interpretation at the segment level

Equation (9) can be rewritten as (see details in Appendix 1)


(13) $$\begin{array}{l} M_{v}^{T}=\frac{\sum_{k=1}^{K}w_{vk}^{T}M_{vk}^{\mu }}{\sum_{k=1}^{K}w_{vk}^{T}}\\ \;=\frac{\sum_{s}l_{vs}M_{vs}}{\sum_{s}l_{vs}}, \end{array}$$

which states that the tract-specific map in a voxel *v* results from the contributions of all streamline segments in a specific voxel. Each segment contributes its segment-specific microstructural metric $M_{vs}^{T}$ defined in Equation (5), weighted by its intra-voxel length *l*_*vs*_. The quantity is normalized by the total segment length in that voxel.

Similarly, Equation (10) using Equation (11) can be rewritten as (see Appendix 1)


(14) $$\begin{array}{l} {\bar{M}}^{T}n=\frac{\sum_{v}\gamma_{v}^{T}M_{v}^{T}}{\sum_{v}\gamma_{v}^{T}}\\ \;=\frac{\sum_{v}\sum_{s}l_{vs}M_{vs}}{\sum_{v}\sum_{s}l_{vs}}, \end{array}$$

where the interpretation is similar to the tract-specific microstructure map $M_{v}^{T}$ above, except for the contributions which are from all segments over all the voxels containing streamlines of tract T.

## 3. Materials and methods

### 3.1. Datasets

Our proposed framework and angular weighting strategy were validated using three datasets: a synthetic phantom, a scan and rescan on a healthy adult volunteer and cohorts of dyslexic children and control children. The synthetic phantom provided a comparison of the different approaches to a known ground truth. The scan and rescan enabled an analysis of the variability and reproducibility of the results. Lastly, the dyslexic cohort served as proof of concept that our framework could be applied to clinical populations.

#### 3.1.1. Experiment I: synthetic phantom

A synthetic phantom based on Monte Carlo simulations of the dMRI signal (Hall and Alexander, 2009; Rensonnet et al., 2015, 2018) was created to compare the microstructural metrics obtained to a known ground truth. The dMRI protocol used for the phantom matched as closely as possible the protocol used in the *in vivo* acquisitions described below. Axons were modeled as straight, randomly-packed cylinders with diameters drawn from a gamma distribution with mean and variance fixed to 1.0μm and 0.6μm, respectively (Rensonnet et al., 2015). The cylinder packing density was interpreted as a fiber volume fraction (FVF). Intra-axonal diffusivity was fixed to 2.0μm^2^ (Dhital et al., 2019). As visible in Figure 4, the phantom was a 2D slice containing four tracts: two horizontal and two vertical tracts. The top and bottom tracts had a FVF of 0.70 and 0.66, respectively, while the vertical tracts had an increasing FVF from top to bottom. All voxels had an extracellular diffusivity $D_{\text{ex}}=1.0{\mu \text{m}}^{2}$. Each vertical tract crossed both horizontal tracts over multiple voxels. Regions of isotropic diffusion representing cerebrospinal fluid (CSF) were also included.

#### 3.1.2. Experiment II: scan and rescan

A healthy adult participant underwent two consecutive dMRI scans to study the variability in the outputs of our method. The scans were performed on a 3T GE SIGNA Premier scanner (GE Healthcare, Chicago, IL) with the following parameters: TR = 4,842 ms, TE = 77 ms, 2 mm isotropic voxels, in-plane FOV: 220 × 220 mm^2^, Δ = 35.7 ms, δ = 22.9 ms, 64 gradients at *b* = 1,000, 32 at b = 2,000, 3,000, 5,000 s/mm^2^, corresponding to diffusion gradient intensities up to 68.9 mT/m, and 7 interspersed b0 images. Preprocessing included thermal denoising (Veraart et al., 2016), Gibbs ringing correction (Kellner et al., 2016), eddy-current distortion and movement correction (Andersson and Sotiropoulos, 2016). The movement correction procedure provided variables representing the relative movement of the patient during the scan time (Bastiani et al., 2019). The total relative motion, representing the average voxel displacement across all voxels with respect to the previous volume, for all volumes, was selected as a summary measure *X*_mov_ of the patient's movement. A 3D T1 image (TE = 2.96 ms, TR = 2188.16 ms, TI = 900 ms, 156 slices, 1 mm isotropic, in-plane FOV: 256 × 256 mm^2^) was also acquired with each scan. Registration to the Desikan-Killiany atlas (Desikan et al., 2006) was accomplished using the *FreeSurfer*^1^ function recon-all, with an additional parcellation of the brainstem. Differences in all tract-specific metrics were computed between the scan and the rescan in 38 major white matter pathways (see methodological details in Section 3.2.2).

#### 3.1.3. Experiment III: dyslexia study

The study consisted of 16 children with dyslexia, a reading and spelling disorder, and 18 healthy controls in the same age range (9.5 ± 1 years old). The experiment was carried out with respect to the ethical standards of the Declaration of Helsinki and received approval by the Ethics Committee of the University Hospital of Saint-Luc (number: B403201942022). All participants underwent a dMRI sequence with the same parameters as in Experiment II above. The registration also used the *FreeSurfer* parcellation. Two macroscopic WM tracts of interest were selected to compare the two populations: the right arcuate fasciculus (AF) and the right superior longitudinal fasciculus II (SLFII). These tracts were selected for their involvement in the language-related pathways and, potentially, dyslexia (Vandermosten et al., 2012a,b; Banfi et al., 2019; Vander Stappen et al., 2020). For each average microstructural metric *M* in each tract and for each of the analysis methods described below, the following regression model was estimated


(15) $$\begin{array}{l} M=\beta_{0}+\beta_{\text{dys}}\cdot X_{\text{dys}}+\beta_{\text{mov}}\cdot X_{\text{mov}}, \end{array}$$

where *X*_dys_∈{0, 1} encodes the participant's group and *X*_mov_ is the aggregate movement metric computed with FSL's motion correction routine (Bastiani et al., 2019). Estimates and *p*-values of β_dys_ were reported to assess the difference between the two populations attributed to dyslexia after correcting for movement during the acquisition.

### 3.2. Data processing and analysis

**Figure 3:**
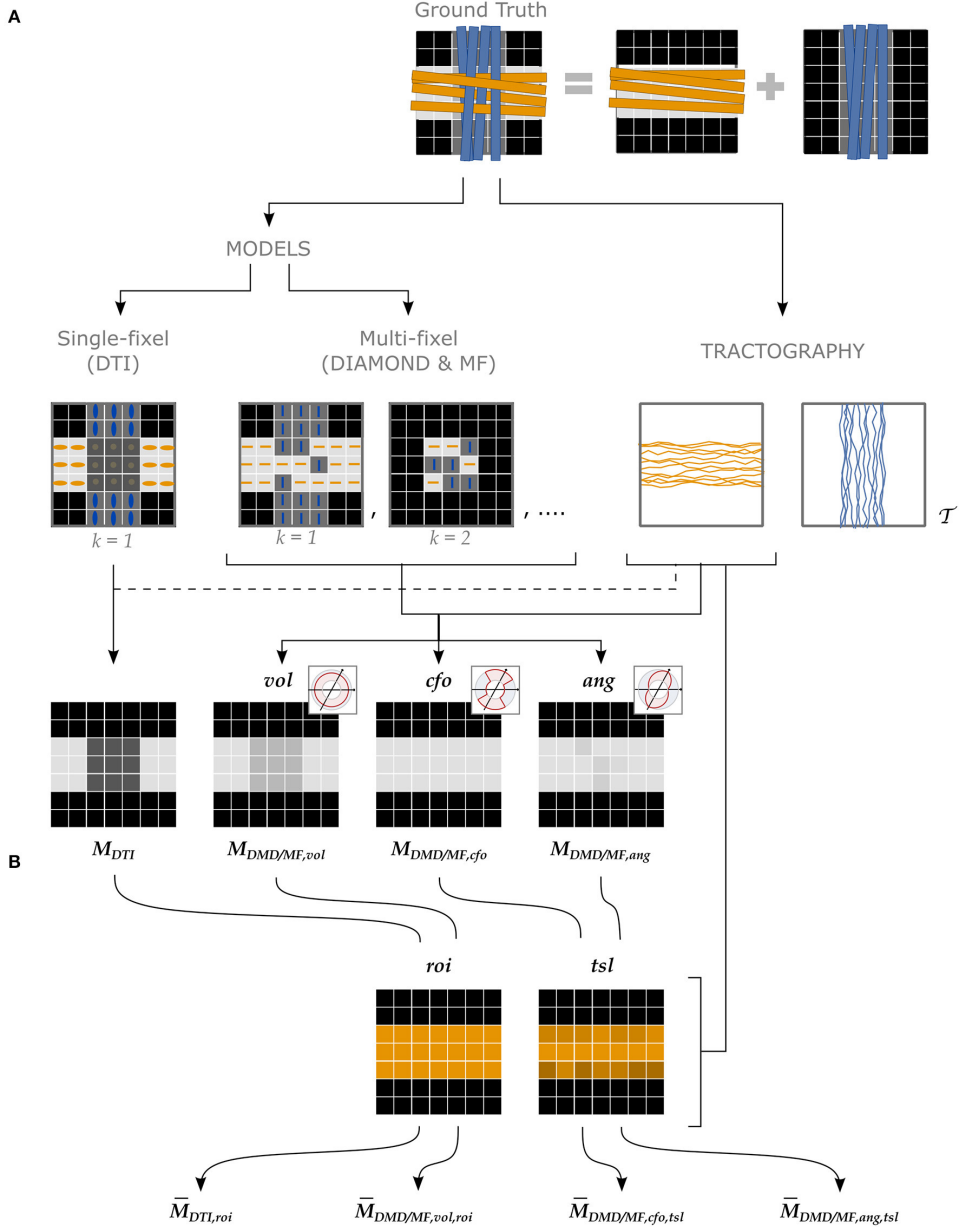
Different approaches to attribute microstructural properties to macroscopic tracts with our proposed UNRAVEL framework. The ground truth (top row) schematically depicts crossing fascicles of axons (not to scale). The grayscale maps in the background show the value of a tract-specific microstructural metric *M*. Either a single- or a multi-fixel model (with *K* = 2 in our example) is estimated (second row). Note that a multi-fixel model does not guarantee a consistent separation of fixels in regions of crossings. The outputs of the proposed method are: **(A)** Microstructure maps (Equation 9), created for each tract T using a relative contribution defined by either *vol, cfo* or *ang*. **(B)** The maps are then averaged as a single value (Equation 10) with the *roi* or *tsl* option.

Four types of estimates for a microstructural property *M* were obtained following the pipeline depicted in Figure 3, described in more detail in the following paragraphs.

#### 3.2.1. Microstructural diffusion models

As shown in the second row of Figure 3, either a single- or a multi-fixel model was estimated at this stage. DTI (Basser et al., 1994) was selected as the single-fixel model while DIAMOND (DMD) (Scherrer et al., 2016) and Microstructure Fingerprinting (MF) (Rensonnet et al., 2019) served as multi-fixel models. DTI and DIAMOND provided tensor-derived metrics such as the fractional anisotropy (FA) while MF estimated the fiber volume fraction (FVF) of each fixel. An isotropic signal contribution was allowed for the multi-fixel models.

#### 3.2.2. Macroscopic tractography analysis

This stage (second row, right in Figure 3) consisted in generating tractography streamlines specific to a tract of interest T. In our experiments, tractography of the WM was performed using a probabilistic algorithm based on the Constrained Spherical Deconvolution (CSD) model (Tournier et al., 2007) to produce a whole-brain tractogram (Garyfallidis et al., 2014). However, the UNRAVEL framework does not constrain the choice of tractography algorithm. In the phantom of Experiment I, the seeds and target regions were placed at the start and end of each tract. In the *in vivo* Experiments II and III, the seeds were placed inside of a T1-based white matter mask with a density of 8 seeds per voxel. The other selected parameters were: a step size of 1 mm, a stopping criterion of 0.35 on the generalized anisotropy and a maximum angle of 15° between streamline segments. The WM tracts of interest were extracted from the whole brain tractography using White Matter Query Language (WMQL) (Wassermann et al., 2016).^2^

#### 3.2.3. Microstructure maps and scalars

The final stages consisted in computing maps (Figure 3A) and averages (Figure 3B) of the metrics of the tracts of interest. For ease of notation, we denote the microstructure map of a tract T for a metric *M* from a model (either DTI, DMD, or MF) as


(16) $$\begin{array}{l} M_{\text{MODEL},A}^{T}, \end{array}$$

where A∈{cfo,ang,vol} specifies the strategy used to define the relative contribution α_*vsk*_ of a fixel to a streamline segment, as defined in Section 2.1.1. Similarly, the mean of the microstructure map is written as


(17) $$\begin{array}{l} {\bar{M}}_{\text{MODEL},A,C}^{T}, \end{array}$$

where C∈{tsl,roi} specifies the tract-averaging strategy $\gamma_{v}^{T}$ selected as defined in Section 2.1.5.

Although all combinations of A and C are compatible, four cases were selected to showcase the currently-available options and the variety of analysis possible with the UNRAVEL framework, and to serve as a baseline to compare our proposed angular weighting strategy (Figure 3). The commonly-used ROI-based single-fixel analysis was represented by the DTI model with region of interest weighting (*roi*). A similar heuristic approach is developed using multi-fixel output with a relative volume and region of interest weighting (*vol,roi*), where the streamline orientation and density do not have an effect on the final estimate. In contrast, the last two approaches, corresponding to either closest-fixel-only or angular weighting combined with total segment length weighting (*cfo/ang,tsl*), are highly impacted by the tractography with the influence of streamline orientation and density on the estimated mean metric. The closest-fixel-only (*cfo*) strategy is commonly used when assigning multi-fixel microstructural properties to streamlines (Raffelt et al., 2015; Rensonnet et al., 2019; Reymbaut et al., 2021) and serves as a baseline for the proposed angular weighting (*ang*) approach.

For all three experiments, we reported estimates of the FA obtained with the four approaches using the FA from DTI and the FA of the diffusion tensors found by DIAMOND (DMD) and named ${\text{FA}}_{\text{DTI}}^{T}$, ${\text{FA}}_{\text{DMD},vol}^{T}$, ${\text{FA}}_{\text{DMD},\text{cfo}}^{T}$ and ${\text{FA}}_{\text{DMD},ang}^{T}$. Metrics obtained with DTI do not specify the α_*vsk*_ used since all three definitions attribute all the weight to the only fixel present. The FVF was only obtained with the last three approaches, corresponding to ${\text{FVF}}_{\text{MF},\text{vol}}^{T}$, ${\text{FVF}}_{\text{MF},\text{cfo}}^{T}$ and ${\text{FVF}}_{\text{MF},\text{ang}}^{T}$, since this variable was only available with MF.

## 4. Results

### 4.1. Experiment I: synthetic phantom

**Figure 4:**
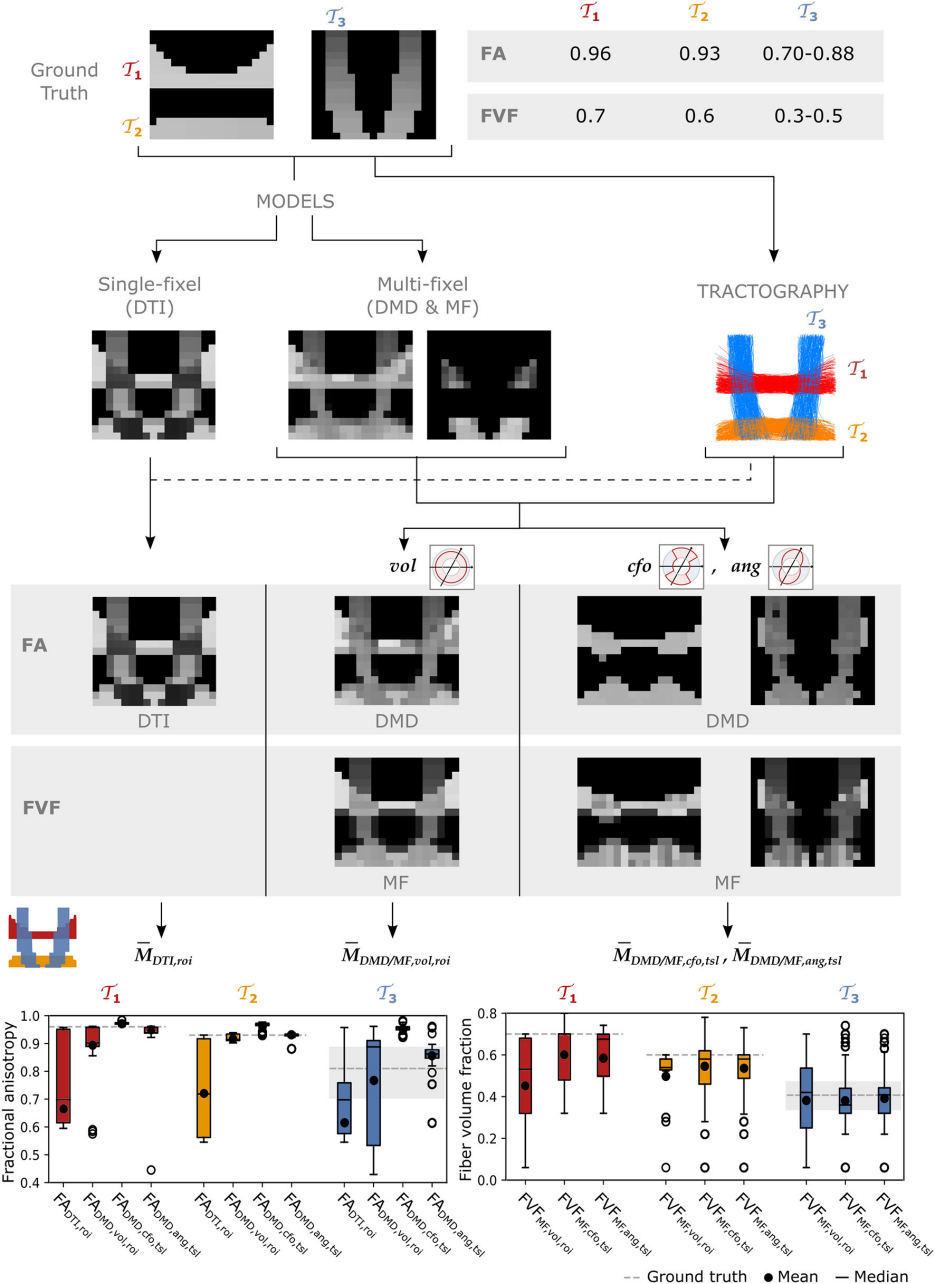
The UNRAVEL framework enables more accurate estimation of the tract-specific microstructure, less impacted by tract crossings. Two horizontal tracts $T_{1}$ and $T_{2}$ with a high FA and FVF are crossed by vertical tracts $T_{3}$ with lower FA and FVF. Tract-specific microstructure maps, defined by Equation (9), are shown for the microstructural metrics FA and FVF. Bottom row: the mean (circle), median (dash), and interquartile range (boxes) of FA and FVF values found for each tract are displayed, the average ground truth value is indicated by a continuous gray line while the minimum and maximum values are shown by dashed gray lines.

Figure 4 reports the tract-specific FA and FVF maps found with the different approaches as well as the means, medians, and interquartile ranges for each tract. With the single-fascicle model, *FA*_DTI_ presented a noticeable decrease in areas of crossing fibers between the horizontal and vertical tracts. The variation along all tracts was high, with *FA*_DTI_ ranging from 0.55 to 0.97 in tracts $T_{1}$ and $T_{2}$ where the ground truth value did not vary along the tract. The mean ${\bar{\text{FA}}}_{\text{DTI},roi}$ in all three tracts was lower than the minimum value in the ground truth. With a multi-fixel model and relative volume weighting, the *FA*_DMD, *vol*_ values were closer to the ground truth than the traditional *FA*_DTI_ in all tracts, especially in areas of crossing fascicles. The values still underestimated the ground truth except in tract $T_{3}$ where the median of the *FA*_DMD, *vol*_ estimates exceeded the highest value in the ground truth. The closest-fixel-only approach displayed less variation than *FA*_DTI_ and *FA*_DMD, *vol*_, but overestimated the *FA*_DMD, *cfo*_ values. Our proposed *FA*_DMD, *ang*_ values also displayed less variation than *FA*_DTI_ and *FA*_DMD, *vol*_ but was closer to the ground truth than *FA*_DMD, *cfo*_. Estimates of *FVF*_MF, *cfo*_ and *FVF*_MF, *ang*_ were similar and presented less variation compared to *FVF*_MF, *vol*_, except for tract $T_{2}$ where *FVF*_MF, *ang*_ exhibited a larger variation. The values of the *FA*_DMD, *ang*_ and *FVF*_MF, *ang*_ microstructure maps displayed in Figure 4 should be interpreted with caution along the edges of the tracts as a low number of streamline segments were contained in those voxels.

**Figure 5:**
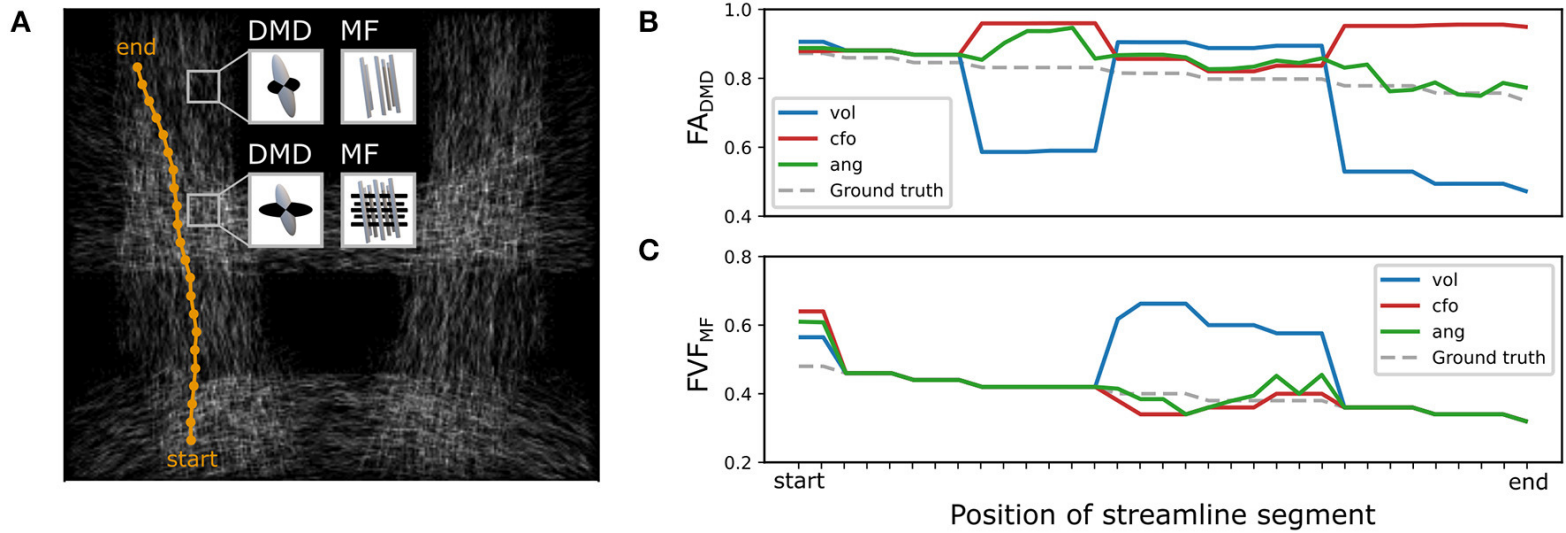
Angular-weighted relative fixel contribution robustly captures varying microstructure along the course of a single streamline. **(A)** A single streamline was isolated (in orange) and all its segments were investigated. The local multi-fixel models were **(B)** DIAMOND (DMD) and **(C)** Microstructure Fingerprinting (MF), each leading to different fixel orientations in each voxel. The DMD model incorrectly estimated two populations in the top half of the vertical tracts. The **(B)** FA or **(C)** FVF values attributed to the streamline segments were computed from the FA or FVF of the multiple fixels in the voxel, following Equation (5). For both **(B, C)**, the values were estimated using the relative volume weighting approach (Equation 2, in blue), closest-fixel-only approach (Equation 3, in red) and the angular weighting approach (Equation 4, in green).

The differences between closest-fixel-only and angular weighting were investigated in Figure 5, which shows the evolution of the segment-specific microstructural metrics FA, from DIAMOND, and FVF, from MF, assigned to each segment of a single streamline isolated from tract $T_{3}$ (Figure 5A), as defined by Equation (5), using relative volume weighting (Equation 2), closest-fixel-only (Equation 3) and angular weighting (Equation 4). In Figure 5B, DIAMOND incorrectly detected two different fixels in the voxels of the upper part of tract $T_{3}$ whereas the ground truth only contained one fixel. The erroneous fixel most aligned with the streamline exhibited a FA greater than the ground truth, while the other presented a lower FA. This led to an overestimation of the streamline-specific *FA*_DMD, *cfo*_, whereas there was either less or no impact on *FA*_DMD, *ang*_, which enables multiple fixels to contribute to a streamline segment. In Figure 5C, with the orientations used by MF, the closest-fixel-only and angular weighting strategies yielded similar estimates of the streamline-specific *FVF*_MF_, which gradually decreased from the lower to the upper part of the isolated streamline.

### 4.2. Experiment II: scan and rescan

**Figure 6:**
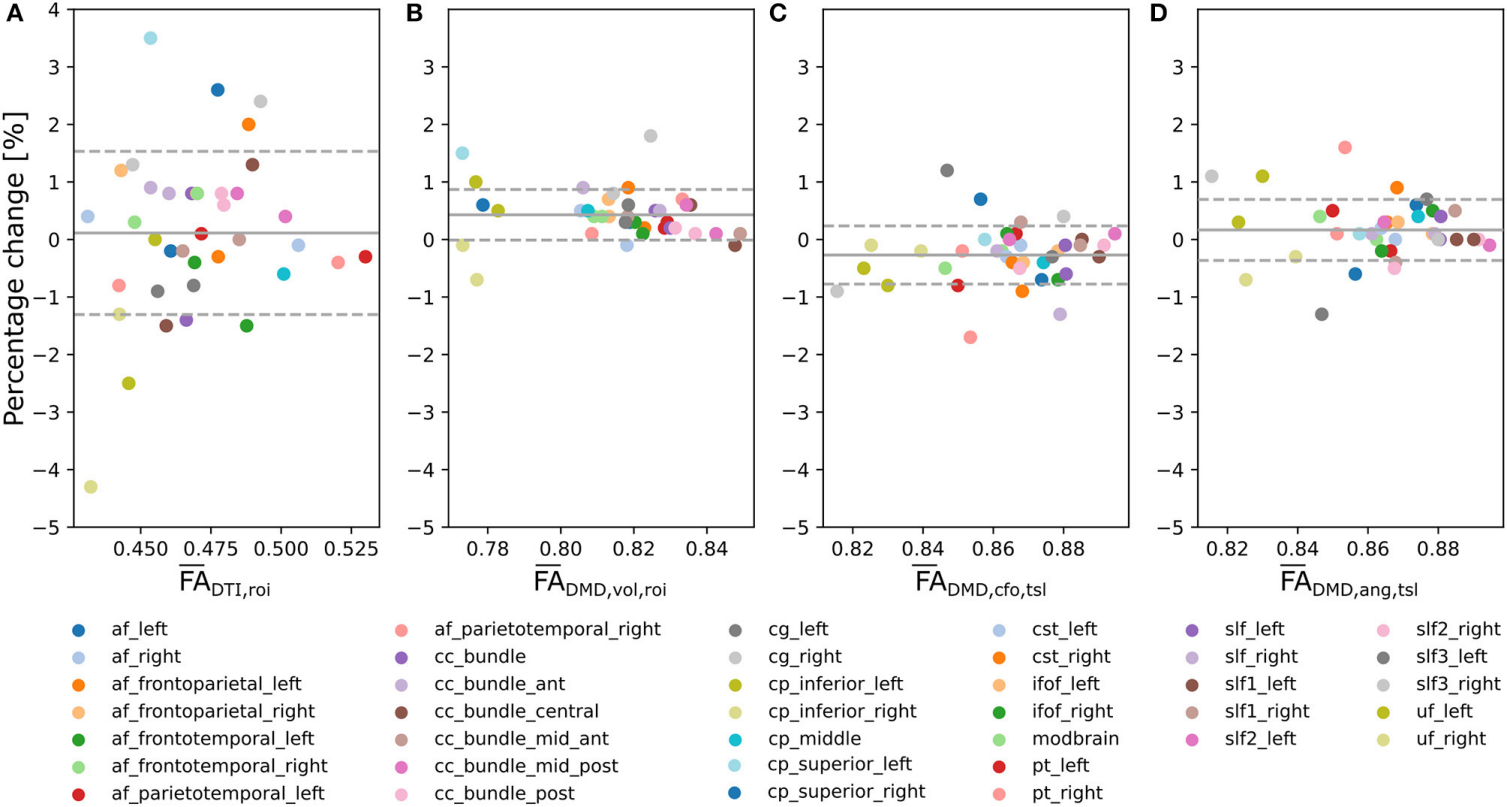
Multi-fixel metrics combined with angular weighting shows smaller variability compared to single-fixel metrics and smaller mean bias compared to relative fraction weighting in a scan/rescan experiment. Bland-Altman plots of the percentage change between the scan and the rescan of, respectively, **(A)** ${\bar{\text{FA}}}_{\text{DTI},roi}$ (Mean = 0.11;SD = 1.4), **(B)** ${\bar{\text{FA}}}_{\text{DMD},vol,roi}$ (Mean = 0.43; SD = 0.44), **(C)** ${\bar{\text{FA}}}_{\text{DMD},\text{cfo},tsl}$ (Mean = −0.27; SD = 0.51) and **(D)** the proposed ${\bar{\text{FA}}}_{\text{DMD},ang,tsl}$ (Mean = 0.17;SD = 0.53) from Equation (10) across the 38 considered WM tracts.

Bland-Altman plots for the tract-wide means ${\bar{\text{FA}}}_{\text{DTI},roi}$ (Figure 6A), ${\bar{\text{FA}}}_{\text{DMD},vol,roi}$ (Figure 6B), ${\bar{\text{FA}}}_{\text{DMD},\text{cfo},tsl}$ (Figure 6C), and our proposed average ${\bar{\text{FA}}}_{\text{DMD},ang,tsl}$ (Figure 6D) defined by Equation (10) for each of the 38 selected tracts suggest smaller changes between the scan and the rescan for ${\bar{\text{FA}}}_{\text{DMD},vol,roi}$, ${\bar{\text{FA}}}_{\text{DMD},\text{cfo},tsl}$, and the proposed ${\bar{\text{FA}}}_{\text{DMD},ang,tsl}$ than for ${\bar{\text{FA}}}_{\text{DTI},roi}$ across the 38 WM regions. Tracts with a higher mean FA showed less variation between the two scans across all approaches. The mean percentage change of ${\bar{\text{FA}}}_{\text{DMD},ang,tsl}$ was closer to zero compared to ${\bar{\text{FA}}}_{\text{DMD},vol,roi}$ and ${\bar{\text{FA}}}_{\text{DMD},\text{cfo},tsl}$. The tract-wide means were higher for ${\bar{\text{FA}}}_{\text{DMD},\text{cfo},tsl}$ and ${\bar{\text{FA}}}_{\text{DMD},ang,tsl}$ than for ${\bar{\text{FA}}}_{\text{DMD},vol,roi}$, all being considerably higher than the traditional ${\bar{\text{FA}}}_{\text{DTI},roi}$.

**Figure 7:**
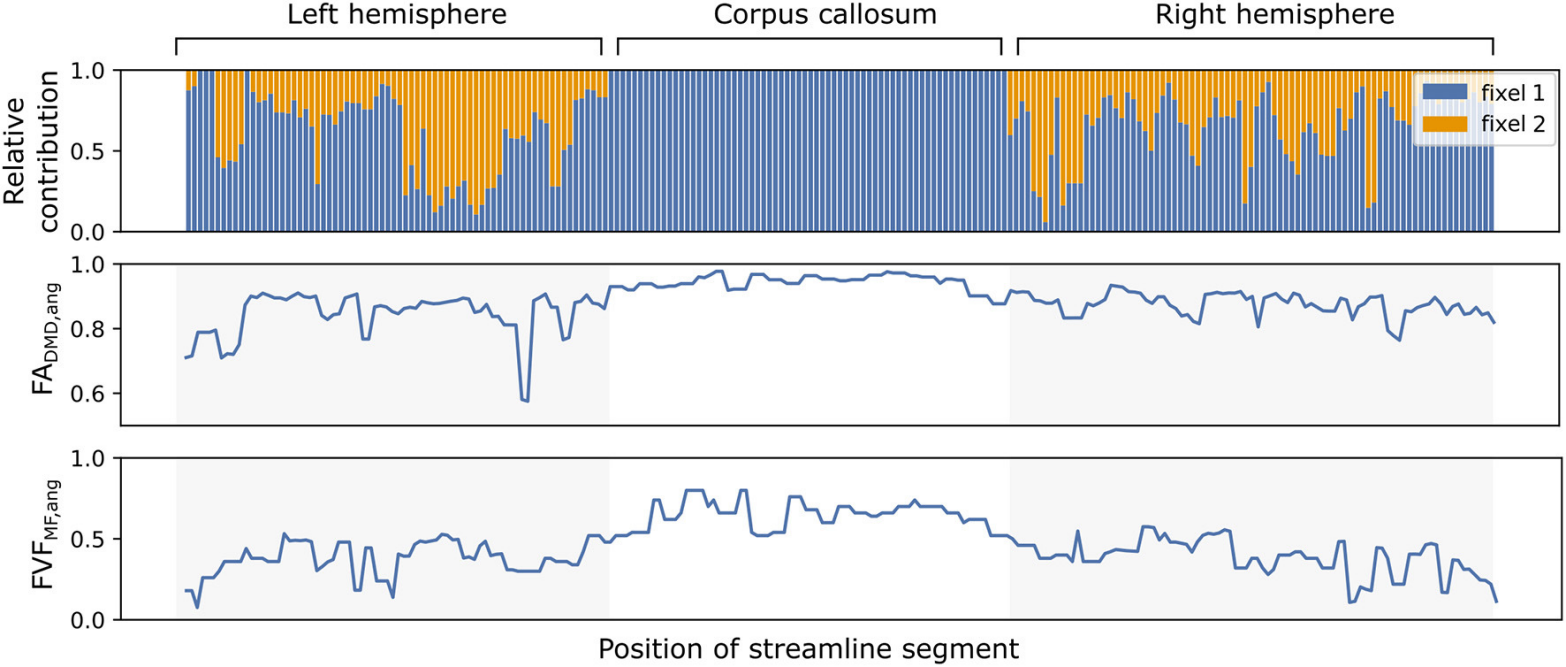
The microstructure along a streamline follows macrostructural changes through brain regions with different neural fiber configurations. The evolution of the relative contributions α_*vsk*_ of two fixels (in blue and orange) for a single callosal streamline along its path **(top)**. Segment-specific *FA*_*vs*_ **(middle)** and *FVF*_*vs*_ **(bottom)** values computed with the UNRAVEL framework using Equation (5).

Similarly to Experiment I, the evolution of the microstructural properties along the path of a single streamline can be obtained with *in vivo* tracts. Figure 7 displays the relative contribution α_*vsk*_ using angular weighting (Equation 4) as well as the associated metrics *FA*_DMD, *ang*_ and *FVF*_MF, *ang*_ for an isolated streamline passing through the corpus callosum. The neural fibers in the middle of the pathway, linking the left and right hemispheres, presented a high *FA*_DMD, *ang*_ and *FVF*_MF, *ang*_, were well-aligned and accurately represented by a single fiber population. The end and start of the pathway displayed lower *FA*_DMD, *ang*_ and *FVF*_MF, *ang*_ with smaller relative contributions and weights.

### 4.3. Experiment III: dyslexia study

**Figure 8:**
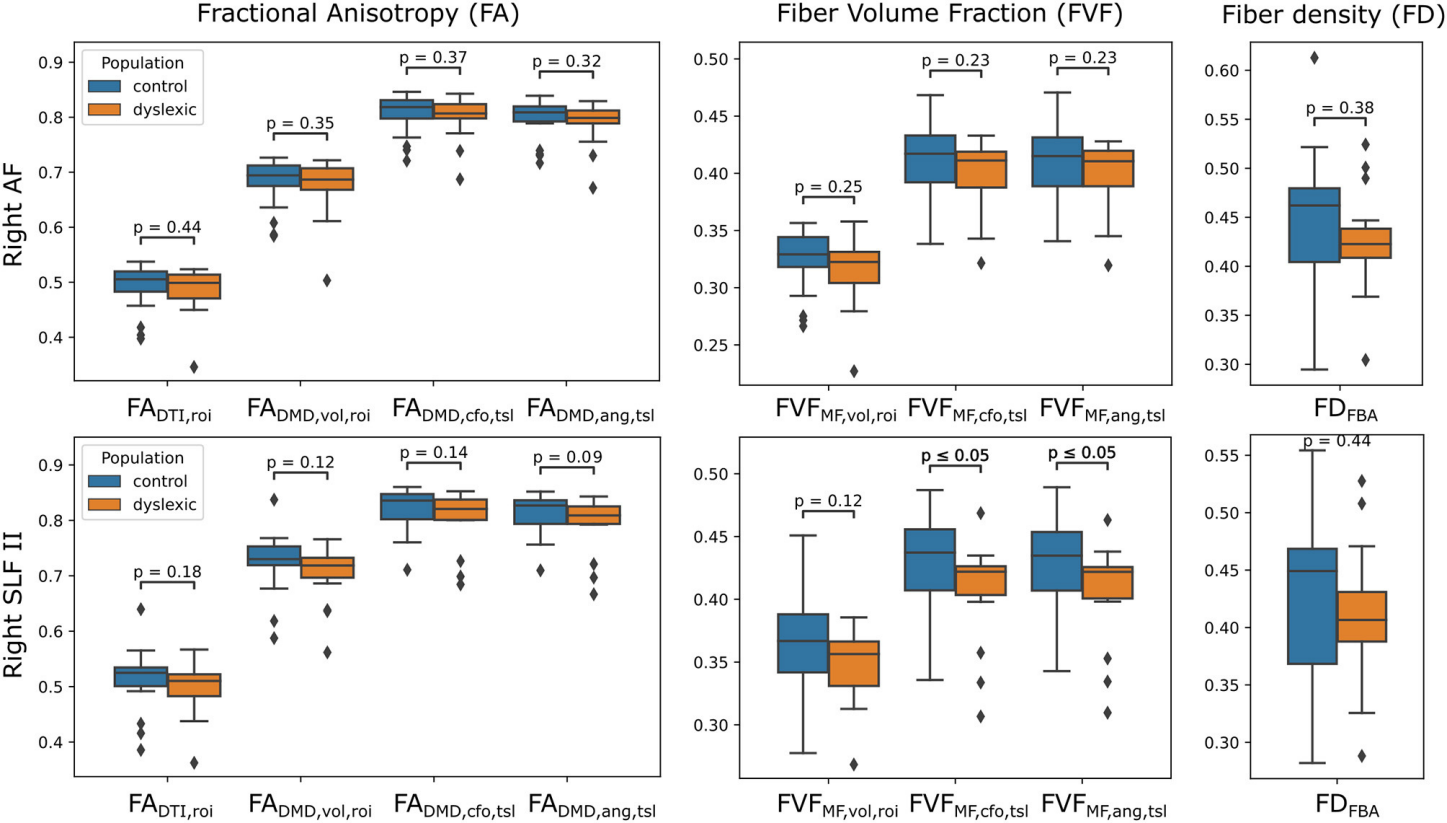
Estimates of FA and FVF obtained with the UNRAVEL framework suggest values are slightly lower in children with dyslexia compared to controls. Boxplots of the tract-wide mean of the fractional anisotropy (${\bar{\text{FA}}}_{\text{DTI}}$, ${\bar{\text{FA}}}_{\text{DMD},vol}$, ${\bar{\text{FA}}}_{\text{DMD},cfo,tsl}$, ${\bar{\text{FA}}}_{\text{DMD},ang,tsl}$), fiber volume fraction (${\bar{\text{FVF}}}_{\text{MF},vol}$, ${\bar{\text{FVF}}}_{\text{MF},cfo,tsl}$, ${\bar{\text{FVF}}}_{\text{MF},ang,tsl}$) and the mean of the fiber density maps obtained with the FBA pipeline *FD*_*FBA*_ for the dyslexic (orange) and control (blue) cohort in the right arcuate fasciculus (AF, **top**) and the right superior longitudinal fasciculus II (SLFII, **bottom**).

The distributions of tract-wide microstructural means for the dyslexic and control populations are shown in Figure 8. The distribution of tract-wide mean FA values (${\bar{\text{FA}}}_{\text{DTI,roi}}$, ${\bar{\text{FA}}}_{\text{DMD},vol,roi}$, ${\bar{\text{FA}}}_{\text{DMD},\text{cfo},tsl}$, ${\bar{\text{FA}}}_{\text{DMD},ang,tsl}$) in each group showed the same behavior, with ${\bar{\text{FA}}}_{\text{DMD},\text{cfo},tsl}$ and the proposed ${\bar{\text{FA}}}_{\text{DMD},ang,tsl}$ having the highest values, followed by ${\bar{\text{FA}}}_{\text{DMD},vol,roi}$ and then by ${\bar{\text{FA}}}_{\text{DTI,roi}}$. The same ordering was observed for ${\bar{\text{FVF}}}_{\text{MF},\text{cfo},tsl}$, ${\bar{\text{FVF}}}_{\text{MF},ang,tsl}$ and ${\bar{\text{FVF}}}_{\text{MF},vol,roi}$ . The mean of the distribution of tract-wide mean FA and FVF was lower in the dyslexic population compared to healthy controls in all cases. Two p-values were below 0.05 when comparing the two groups, ${\bar{\text{FVF}}}_{\text{MF},\text{cfo},tsl}$ and ${\bar{\text{FVF}}}_{\text{MF},ang,tsl}$ in the right SLFII. No statistically-significant differences were found using the fiber density (FD) metric from the FBA framework.

**Figure 9:**
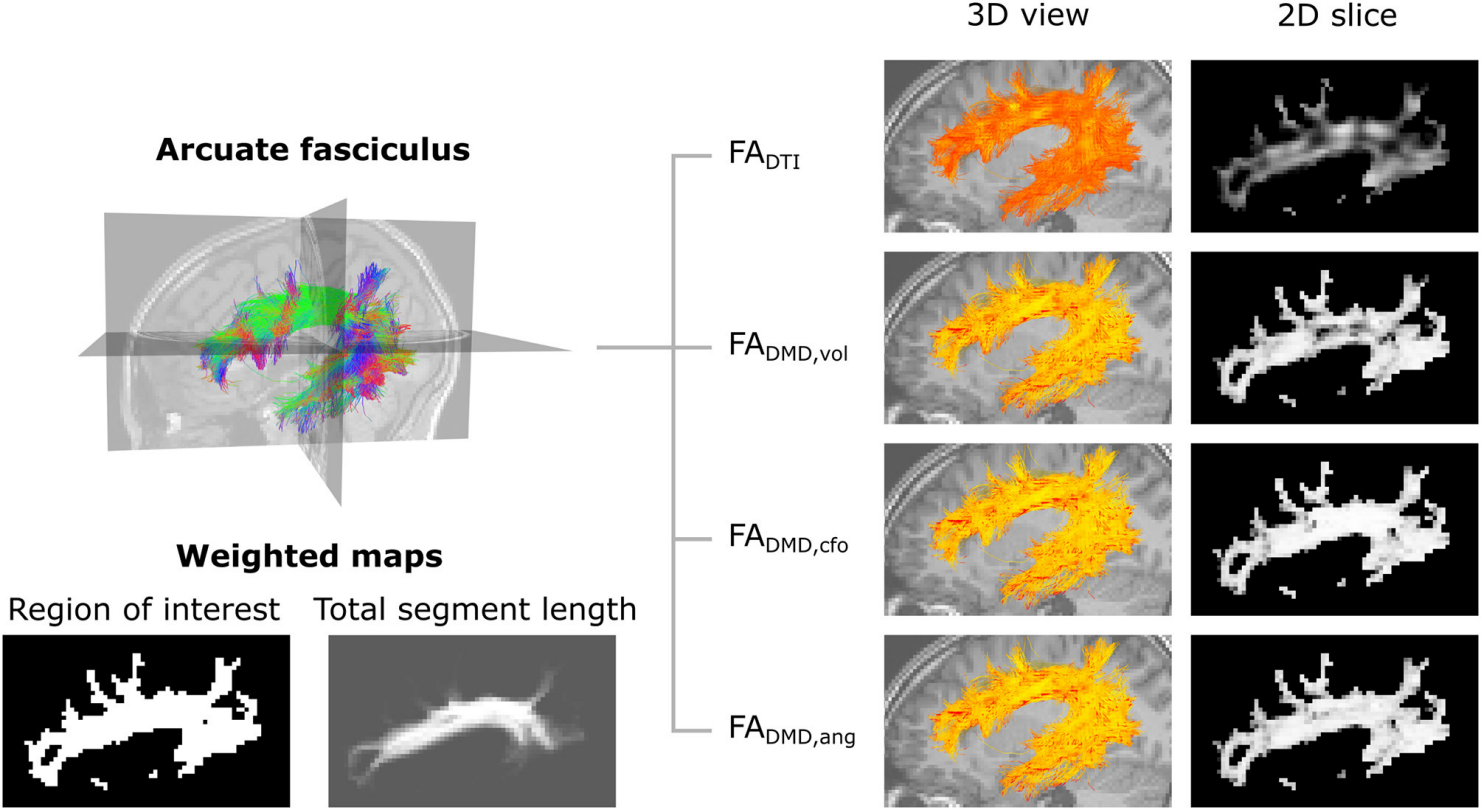
Metrics maps obtained with angular weighting are less impacted by the properties of crossing fibers. **Top-left**: representation of the streamlines of the left arcuate fasciculus tract, color-coded for orientation. **Bottom-left**: weighted maps. The tract-specific total segment length map was obtained with Equation (8) and corresponds to the total length of segments belonging to the AF in each voxel. **Right**: visualization of the microstructure map over a set of 3D streamlines and a 2D slice of the fractional anisotropy obtained with DTI (*FA*_DTI_) and multi-fixel models with: relative volume weighting [*FA*_DMD, *vol*_, see Equation (2)], closest-fixel-only [*FA*_DMD, *cfo*_, see Equation (3)] and our proposed angular weight [*FA*_DMD, *ang*_, see Equation (4)].

Figure 9 displays maps of *FA*_DTI_, *FA*_DMD, *vol*_, *FA*_DMD, *cfo*_ and *FA*_DMD, *ang*_ on a slice of the AF in a control participant. The traditional *FA*_DTI_ values obtained with DTI are lower overall and present several dark spots in areas where the AF fibers are crossing other neural fibers. The *FA*_DMD, *vol*_ map shows higher values and fewer dark spots, while the *FA*_DMD, *cfo*_ and *FA*_DMD, *ang*_ maps show an even more uniform FA map and the dark areas have nearly all disappeared. The total segment length map $w_{v}^{T}$, obtained with Equation (8), shows the attribution of a higher weight to voxels in the center part of the AF compared to voxels on the edges.

**Figure 10:**
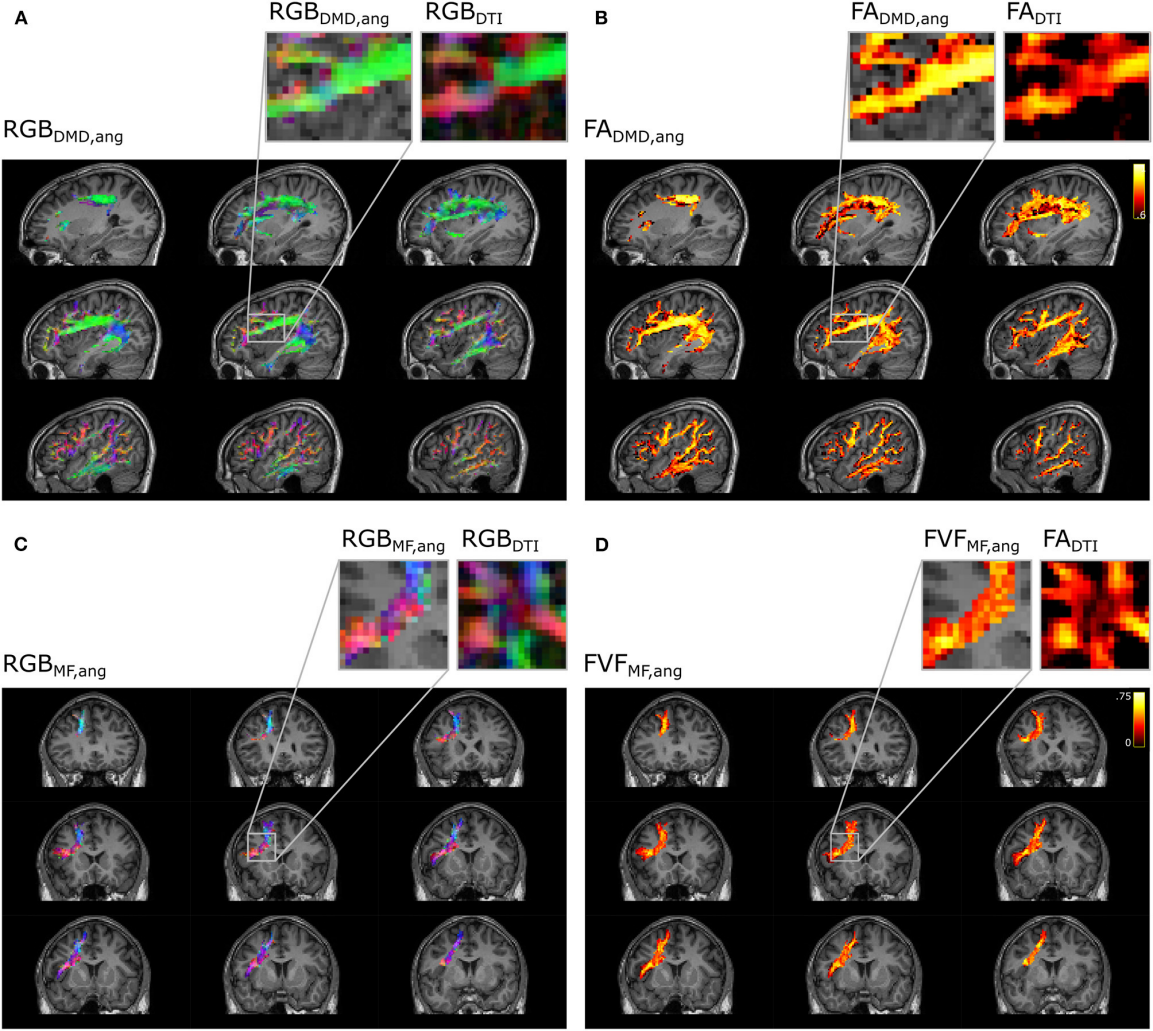
Metrics maps using angular weight recover the properties along the direction of the tract. Microstructure maps of the arcuate fasciculus **(A, B)** and the frontal aslant tract **(C, D)**. **(A)** Color-coded maps (RGB) of the orientation of the fixel obtained with DIAMOND (DMD) and **(C)** Microstructure Fingerprinting (MF) are compared to maps obtained with DTI. Microstructural maps of the **(B)** FA and **(D)** FVF are also compared to the FA obtained with DTI.

The microstructural maps obtained with multi-fixel models combined with angular weighting displayed in Figure 10 show a more uniform orientation in areas of crossing fibers compared to the maps obtained with the single-fixel model DTI. The AF (Figures 10A, B) is predominantly aligned in the antero-posterior direction (green) in the area where it crosses the frontal aslant tract (FAT), whereas maps obtained with DTI present more diverging orientations and a dominant left-right (red) orientation in several voxels. The *FA*_DTI_ obtained was also lower in areas of crossing fibers. The FAT shows similar results (Figures 10C, D) where it crosses the corpus callosum connections, with a gain in directionality and a more coherent FVF measure compared to DTI indices.

## 5. Discussion

### 5.1. Accuracy of the estimation of tract-specific microstructure

When comparing the closest-fixel-only and the proposed angular weighting strategies with relative volume weighting in multi-fixel metrics and with the single-fixel metrics, we observed differences in FA and FVF across all three datasets (Figures 4, 6, 8), due to (i) multi-fixel information being more accurate than single-fixel information and ii) a focus on the fixels aligned with the tract of interest in each voxel, leading to less contamination by crossing fascicles.

In the synthetic phantom (Figure 4), the single-fixel model was inadequate in areas of crossing fibers, as the estimated microstructural properties corresponded to neither of the fiber populations present in the voxel. Using a multi-fixel model capable of discerning the properties of multiple fiber populations in a single voxel, the FA and FVF were still underestimated with relative volume weighting (*vol*) in the horizontal tracts with high ground truth values. Showing that a macroscopic analysis that does not take into account the orientation of the microstructural fixels leads to a sub-optimal estimation of the underlying microstructure. An increased tract-wide mean with angular and total segment length weighting (*ang,tsl*) compared to relative volume and region of interest weighting (*vol,roi*) was also observed in Experiments II and III, which we attribute to the reduction of contamination by crossing fascicles and the use of the total segment length (*tsl*) as a weighted map (Equation 8). Voxels occupied by more tract segments have a larger weight in the tract-wide average, which mirrors a higher probability of belonging to the tract of interest. This was further illustrated in Figures 9, 10, where approaches that did not make use of the streamline direction conflated the microstructural metrics of the other fiber tracts intersecting the AF. In Figure 9, some of the anomalies visible with traditional DTI disappeared in the *FA*_DMD, *vol*_ map. Anomalies remaining with *FA*_DMD, *vol*_ were likely due to the averaging of the main fixel properties with a secondary fixel possessing a different FA. The closest-fixel-only and proposed angular weighting strategies bypass these issues by using the orientations obtained from the tractography to more accurately describe brain structures, without interference from crossing fiber tracts.

### 5.2. Robustness to tractography and multi-fixel estimation errors

UNRAVEL makes full use of multi-fixel information and produces results with less variability than single-fixel metrics. In Experiment II (Figure 6), the decreased percentage change of *FA*_DMD, *vol*_ and *FA*_DMD, *ang*_ compared to traditional DTI indicated a higher reliability of these methods in repeated analyses of the same patient, which is a desirable feature in longitudinal studies.

At the microscopic scale, observing the varying microstructure along the course of a single streamline (Figure 7) showed our framework deals with both “one-to-one” and “one-to-many” correspondence issues between fixels of neighboring voxels. Indeed, the most tract-relevant population switched between the two fixels as their orientations became more aligned with the streamlines of the tract and the method had no issues going from a two-fixel voxel to a single-fixel voxel. The segment-specific *FA*_*vs*_ and *FVF*_*vs*_ values of a streamline obtained with UNRAVEL are also consistent with the known macrostructure of the corpus callosum, with the segments in the middle of the path presenting higher axonal density. In a configuration with two fiber populations per voxel (*K* = 2), the proposed angular weighting (Equation 4) is more robust than the commonly-used closest-fixel-only strategy (Equation 3) when the number of fixels is incorrectly estimated by the local microstructural model, as seen in Figures 4, 5, where an incorrect estimation of the number of fixels in the DIAMOND model led to an overestimation of the microstructural metric. In addition, areas in which angular weighting computes fixels' relative contributions close to 1/*K* will likely lead to more stable estimates than with closest-fixel-only, as streamline segments would have been attributed the microstructural properties of different fixels in the same voxel based on small differences in orientation. Angular weighting should also provide more accurate results in areas where there are more fiber populations than estimated fixels, as the properties of the undetected fixels will be distributed among the *K* fixels found by the microstructural model, and angular weighting ensures every fixel will have an impact if they are close to the considered orientation.

At the macroscopic scale, the use of total segment length weighting (*tsl*), defined by Equation (11), reduces the impact of outlier and false-positive streamlines on the proposed tract-based metrics, compared to ROI-based means (*roi*), defined in Equation (12). The total segment length map in Figure 9 illustrates this effect, with a reduced weight on the edge of the tract, as well as a weight close to zero in isolated voxels.

### 5.3. Flexibility and usability

The proposed UNRAVEL framework requires little computing power and accepts a wide range of inputs. The choice of a multi-fixel microstructural model is free as long as each fixel has a principal orientation. This means many fixel-specific properties can be investigated, from diffusivity to axon diameter distribution. This is an advantage compared to methods such as FBA (Raffelt et al., 2017) which focus on axon fiber density and bundle cross-section, COMMIT (Daducci et al., 2015; Schiavi et al., 2020; Ocampo-Pineda et al., 2021) and AxTract (Girard et al., 2017) which require an axon diameter estimation. With our framework, the input streamlines can be generated by any tractography method and segmented into tracts of interest by any approach, from manual to (semi-) automated (Wassermann et al., 2016; Warrington et al., 2020). Another important degree of freedom is the definition of the relative contribution of a fixel to a streamline segment α_*vsk*_ and weighted map $\gamma_{v}^{T}$. Besides the definitions presented in Section 2.1, additional weighting strategies can be defined and included in our framework. In cases where tractography is not available, the tract-specific microstructure maps obtained with relative volume weighting (*vol*) can be used to perform traditional voxel-based analysis (VBA) and region-based analysis using the region of interest weighting (*roi*) by supplying a ROI as input instead of a tract of interest, since relative volume weighting is not dependent on the angular information contained in the tractography, as shown in Figure 2. Finally, our framework enables analysis at different scales: microstructural properties can be obtained for individual streamlines via Equation (5) as in Figures 5, 7, as well as volumetric maps specific to a tract via Equation (9) as in Figures 4, 9, 10, and tract-wide summary metrics via Equation (10) as in Figures 6, 8.

### 5.4. Limitations

The UNRAVEL framework is affected by errors in its two inputs and may propagate those estimation errors. Multi-fixel models may incorrectly characterize the fiber populations in a voxel, as in Experiment I (Figure 5). However, the choice of angular weighting (Equation 4) was shown to reduce this impact. Additionally, total segment length weighting was found to reduce the variability of probabilistic tractography, as seen in Figures 5, 9. Nonetheless, the UNRAVEL framework is affected by noise from local estimates, resulting in noisy estimates as in the FVF maps in Figure 4. To address this limitation, spatial regularization across fixels belonging to similar macroscopic tracts could be implemented, as proposed in Raffelt et al. (2015).

Another limitation of our experiments is the restriction of multi-fixel models to only two fixels per voxel (*K* = 2), which is known to be insufficient in regions such as the centrum semiovale where the corticospinal tract, fibers from the corpus callosum, and the superior longitudinal fasciculus intersect. This decision was made due to the challenges in achieving a stable and robust fit for complex multi-fixel models with up to three fixels using current clinical dMRI acquisitions. Other relative contribution definitions might be more suitable when *K*>2, as the relative weights of aligned fixels will decrease as K increases.

The WM tracts analyzed in our *in vivo* experiments were limited to long-range main white matter pathways, and did not include short-range WM fibers connecting neighboring cortical areas, known as U-fibers. However, the analysis of such fibers using UNRAVEL should not raise issues beyond the need for an accurate tractogram and model estimation as inputs. Furthermore, since the UNRAVEL framework is not dependent on a single model or tractography algorithm, any future improvements in the accuracy of either input will be compatible with our framework and lead to more accurate results.

Finally, although changes in *FA*_DMD, *ang*_ and *FVF*_MF, *ang*_ suggested the same trend, few statistically significant differences between control and dyslexic children were found in Experiment III. However, this might be due to the restricted sample size and small effect size, which is a well-known pitfall of neuroimaging studies in psychology and psychiatry (Button et al., 2013; Thompson et al., 2020). Nevertheless, we demonstrated the feasibility of applying our framework to clinical populations and the consistency of the metrics obtained with our approach.

## 6. Conclusion

In this study, we have introduced UNRAVEL, a framework combining the macrostructural information of tractography with the microstructural metrics of multi-fixel models. Combining these two scales with the proposed angular weighting strategy allows tract-specific analyses to be less impacted by crossing fiber tracts, while retaining some robustness in case of erroneous tractography or diffusion model estimations. We demonstrated the feasibility of our framework and the accuracy of our angular weighting algorithm both on synthetic and *in vivo* data. The UNRAVEL framework will provide researchers in the medical field and the diffusion MRI community with a flexible tool to study, visualize and more easily interpret the microstructure of macroscopic white matter pathways in individual cases as well as population studies.

## Data availability statement

Publicly available datasets were analyzed in this study. This data can be found here: the code is open source and freely available at https://github.com/DelinteNicolas/UNRAVEL (https://www.doi.org/10.5281/zenodo.7753501). The programming language is Python and the package is platform independent. The brain MRI data was obtained in the Brussels Saint-Luc University Hospital, Belgium. The data of the adult participant is available from the corresponding author upon reasonable request. The data related to the dyslexic and control children is not available due to privacy issues of clinical data.

## Ethics statement

The studies involving human participants were reviewed and approved by the experiment was carried out with respect to the ethical standards of the Declaration of Helsinki and received approval by the Ethics Committee of the University Hospital of Saint-Luc (number: B403201942022). Written informed consent to participate in this study was provided by the participants' legal guardian/next of kin.

## Author contributions

ND and GR conceived the presented idea and developed the theory. CG, MV, and LD participated in the data collection. BM and GR supervised the project. All authors contributed to the manuscript revision, read, and approved the submitted version.

## Footnotes

^1^ https://surfer.nmr.mgh.harvard.edu/

^2^The WMQL queries used are available on the GitHub page of our project at https://github.com/DelinteNicolas/UNRAVEL.

## Appendix

### A. Interpretation at the segment level

This section provides another perspective on the microstructure maps (Equation 9) and the mean tract microstructural metric (Equation 10), interpreted at the level of individual streamline segments.

Equation (9) can be rewritten as follows

(A1)
$$\begin{array}{l} M_{v}^{T}=\frac{\sum_{k=1}^{K}w_{vk}^{T}M_{vk}^{\mu }}{\sum_{k=1}^{K}w_{vk}^{T}}\\ \;=\frac{\sum_{k=1}^{K}\alpha_{vsk}l_{vs}M_{vk}^{\mu }}{\sum_{k=1}^{K}\alpha_{vsk}l_{vs}}\;(*)\\ \;=\frac{\sum_{s}l_{vs}\sum_{k=1}^{K}\alpha_{vsk}M_{vk}^{\mu }}{\sum_{s}l_{vs}\sum_{k=1}^{K}\alpha_{vsk}}\;(**)\\ \;=\frac{\sum_{s}l_{vs}\sum_{k=1}^{K}\alpha_{vsk}M_{vk}^{\mu }}{\sum_{s}l_{vs}}\;(***)\\ \;=\frac{\sum_{s}l_{vs}M_{vs}}{\sum_{s}l_{vs}},\;(****) \end{array}$$

where (*) uses Equation (7), (**) switches the order of summation, (***) uses Equation (1) and (****) uses Equation (5). The last equality states that the tract-specific map in a voxel *v* results from the contributions of all streamline segments in that voxel. Each segment contributes its segment-specific microstructural metric defined in Equation (5), weighted by its intra-voxel length *l*_*vs*_. The quantity is normalized by the total segment length in that voxel.

Similarly, Equation (10) using Equation (11) can be rewritten as

(A2)
$$\begin{array}{l} {\bar{M}}^{T}=\;\frac{\sum_{v}\gamma_{v}^{T}M_{v}^{T}}{\sum_{v}\gamma_{v}^{T}}\\ \;=\frac{\sum_{v}\gamma_{v}^{T}\frac{\sum_{s}l_{vs}M_{vs}}{\sum_{s}l_{vs}}}{\sum_{v}\gamma_{v}^{T}}\;(*)\\ \;=\frac{\sum_{v}\sum_{s}l_{vs}\frac{\sum_{s}l_{vs}M_{vs}}{\sum_{s}l_{vs}}}{\sum_{v}\sum_{s}l_{vs}}\;(**)\\ \;=\frac{\sum_{v}\sum_{s}l_{vs}M_{vs}}{\sum_{v}\sum_{s}l_{vs}} \end{array}$$

where (*) uses Equation (13) and (**) uses Equation (8) and Equation (11). The interpretation is similar to the tract-specific microstructure map $M_{v}^{T}$ above, except for the contributions, which are from all segments *s* over all the voxels containing streamlines of tract T.
